# Differentiation of hemispheric white matter lesions in migraine and multiple sclerosis with similar radiological features using advanced MRI

**DOI:** 10.3389/fnins.2024.1384073

**Published:** 2024-05-09

**Authors:** Flóra John, Gréta Kis-Jakab, Hedvig Komáromy, Gábor Perlaki, Gergely Orsi, Edit Bosnyák, Renáta Rozgonyi, Anita Trauninger, Kata Eklics, David Olayinka Kamson, Zoltán Pfund

**Affiliations:** ^1^Department of Neurology, Medical School, University of Pécs, Pécs, Hungary; ^2^HUN-REN-PTE Clinical Neuroscience MR Research Group, Pécs, Hungary; ^3^Department of Neurosurgery, Medical School, University of Pécs, Pécs, Hungary; ^4^Pécs Diagnostic Center, Pécs, Hungary; ^5^Department of Languages for Biomedical Purposes and Communication, University of Pécs, Pécs, Hungary; ^6^Sidney Kimmel Comprehensive Cancer Center at the Johns Hopkins Hospital, Baltimore, MD, United States; ^7^Department of Neurology, Johns Hopkins School of Medicine, Baltimore, MD, United States

**Keywords:** migraine, multiple sclerosis, magnetic resonance spectroscopy, white matter lesions, radiological features

## Abstract

**Background and aim:**

White matter hyperintensities (WMHs), presented on T2-weighted or fluid-attenuated inversion recovery magnetic resonance imaging (MRI) sequences, are lesions in the human brain that can be observed in both migraine and multiple sclerosis (MS).

**Methods:**

Seventeen migraine patients and 15 patients with relapsing–remitting multiple sclerosis with WMHs, and 17 healthy subjects age-and sex-matched to the migraine group were prospectively enrolled and underwent conventional and advanced MRI studies with diffusion-and perfusion-weighted imaging and single voxel proton magnetic resonance spectroscopy.

**Results:**

In both disease groups, elevated T2 relaxation time, apparent diffusion coefficient (ADC) values, and decreased *N*-acetyl-aspartate levels were found in the intralesional white matter compared to the contralateral normal-appearing white matter (NAWM), while there was no difference between the hemispheres of the control subjects. Migraine patients had the lowest intralesional creatine + phosphocreatine and myo-inositol (mI) values among the three groups, while patients with MS showed the highest intralesional T1 and T2 relaxation times, ADC, and mI values. In the contralateral NAWM, the same trend with mI changes was observed in migraineurs and MS patients. No differences in perfusion variables were observed in any groups.

**Conclusion:**

Our multimodal study showed that tissue damage is detectable in both diseases. Despite the differences in various advanced MRI measures, with more severe injury detected in MS lesions, we could not clearly differentiate the two white matter lesion types.

## Highlights

- The locations of MS and migraine intracranial hemispheric lesions are similar.
- Complete separation of MS lesions and migraine lesions using even cutting-edge MRI techniques is difficult. Especially when the MS patient’s EDSS score is ≤3.
- Further difficulties in separation are caused by the effects of oxidative stress and the autoimmunity in MS and migraine.

## Introduction

White matter hyperintensities (WMHs), presented on T2-weighted or fluid-attenuated inversion recovery (FLAIR) magnetic resonance imaging (MRI) sequences, can be observed in several diseases, including hypertension, migraine, multiple sclerosis, and other immune-mediated or hereditary diseases (Porter et al., 2005). Since these WMHs represent non-specific tissue damage in the human brain, we call them white matter lesions (WMLs) later in the text.

Multiple sclerosis (MS) is one of the most common immune-mediated central nervous system (CNS) disorders causing demyelination, inflammation, gliosis, and neuronal loss disseminated in different areas of the CNS and occurring at different times (Thompson et al., 2018). WMLs in MS are typically situated in the juxtacortical, cortical, periventricular, pericallosal, callosal, infratentorial, and spinal cord regions (Thompson et al., 2018). Moreover, contrast-enhancing lesions can also be observed in the active phase, while lesions with severe axonal damage or glial necrosis can be seen as a hypointense region on T1-weighted images (Thompson et al., 2018).

Migraine is a neurological disease characterized by recurrent headache attacks associated with temporary symptoms of autonomic nervous system dysfunction and accompanied by focal auras in some cases (Headache Classification Committee of the International Headache Society (IHS), 2013). Migraine patients have an increased risk of developing supratentorial deep WMLs, or silent posterior ischemic infarcts (Porter et al., 2005). Migraine-related WMLs are small, ovoid lesions mostly found in periventricular and deep brain white matter sparing the juxtacortical region (Kruit et al., 2004). However, in some cases, WMLs in migraine may also be in typical MS areas, such as juxtacortical or callosal regions, and at least 24.4% of headache patients may fulfill the radiological diagnostic (McDonald) criteria for MS (Liu et al., 2013). In addition, the high co-morbidity between MS and migraine is well known (Kister et al., 2010; Villani et al., 2012).

**Figure 1:**
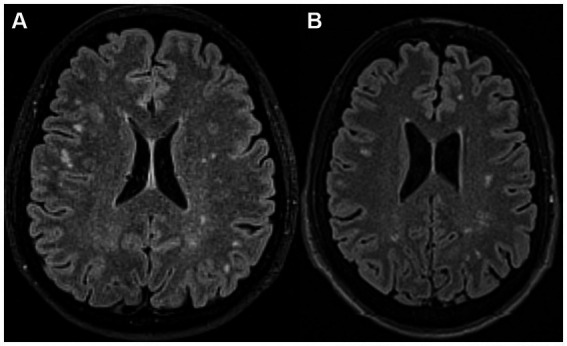
**(A)** A 46-year-old female patient with migraine with aura. The axial fluid-attenuated inversion recovery (FLAIR) MR image shows multiple white matter lesions (WMLs) in both cerebral hemispheres. **(B)** A 32-year-old man with relapsing–remitting multiple sclerosis. The axial FLAIR MR image shows multiple WMLs in both cerebral hemispheres.

Despite the advances in neuroimaging techniques, differentiation of the WMLs of these two pathologies remains difficult (Figure 1). A recent review summarizing proton magnetic resonance spectroscopy (^1^H-MRS) studies in migraine patients concluded that recent studies support the hypothesis of impaired energetics and mitochondrial dysfunction in migraine, showing decreased N-acetyl-aspartate (NAA) and increased lactate levels (Nikolova and Schwedt, 2022). On the other hand, decreased NAA in MS lesions is a commonly reported abnormality among elevated choline (Cho) and myo-inositol (mI) (Heckova et al., 2022; Lipka et al., 2023). However, only a few studies compared WMLs of migraine and MS patients and are mostly confined to diffusion-weighted imaging (DWI) (Orsi et al., 2015; Zacharzewska-Gondek et al., 2019). Our recent study concluded that an accurate differential diagnosis of WMLs by conventional MRI was probably not possible in individual patients (Kamson et al., 2012).

Thus, in this prospective study, we aimed to investigate WMLs in patients with migraine and MS using advanced MRI techniques, such as ^1^H-MRS, perfusion-weighted imaging (PWI), and DWI. Based on the findings, we compared them to a control group to define the differences between the two diseases.

## Patients and methods

### Subjects

Seventeen migraine patients (14 females and 3 males, mean age ± standard deviation [SD]: 42.9 ± 10.6 years, age range: 20–65 years) with previously discovered brain WMLs and 15 patients with relapsing–remitting multiple sclerosis (11 females and 4 males, mean age ± SD: 37.5 ± 8.8 years, age range: 23–51 years) were prospectively enrolled in this study between 2010 and 2017 at the Department of Neurology, Medical School, University of Pécs, Hungary. The most important radiological inclusion criterion was the large lesion size (≥ 7 mm, largest diameter) with normal-appearing white matter (NAWM) on the contralateral side in both groups, and a good physical status of MS patients expanded disability status scale (EDSS) score ≤ 3.

Among the migraine patients, 10 met the International Headache Society classification criteria for migraine without aura and seven for migraine with aura. Migraineurs had only supratentorial WMLs on their T2-weighted and FLAIR MRIs without hypointensity on their T1-weighted MRIs. All patients were only on abortive migraine treatment (such as nonsteroidal anti-inflammatory drugs or triptans) with no chronic prophylactic therapy at the time of the MRI study. The MRI studies were taken during a headache-free period.

All MS patients showed supra-and infratentorial WMLs and were diagnosed with relapsing–remitting MS according to the 2005 modified McDonald criteria (EDSS score ± SD: 1.6 ± 1.53; EDSS range: 0–3), and the diagnosis did not change when we revised these patients with the 2017 McDonald criteria (Thompson et al., 2018). WMLs in MS patients showed no hypointensity on T1-weighted images like the migraineurs, and no contrast enhancement was seen, referring to active demyelination. All patients were on chronic immunomodulatory therapy at the time of the MRI study, and the measurement was retaken in the remission phase.

None of the migraine or MS patients suffered from comorbidities that may cause WMLs, e.g., hypertension, diabetes, thyroid gland disease, other cerebrovascular risk factors, systemic autoimmune diseases. Migraine patients with other types of headaches were also excluded from the study. None of the MS patients had a history of migraine headaches.

As a control group, 17 healthy subjects age-and sex-matched to the migraine group (14 females and 3 males, mean age ± SD: 42.8 ± 10.5 years, age range: 20–65 years) without headache and with a normal MRI were also prospectively enrolled in the study. Patients lacked any comorbidities. Studies were performed in accordance with the approval of the Regional Research Ethics Committee of the Clinical Center, Pécs, and written informed consent was obtained from all study participants.

### MRI scanning protocol and image analysis

MRI was performed on a 3.0-Tesla clinical MRI scanner (Magnetom TIM Trio, Siemens Medical Solutions, Erlangen, Germany), with a field gradient strength of 40 mT/m and a 12-channel phased array head coil. The following MRI sequences were acquired: T1-and T2-weighted and 3-dimensional (3D) FLAIR images, DWI, PWI, ^1^H-MRS, and T1 and T2 relaxation time measurements.

**Figure 2:**
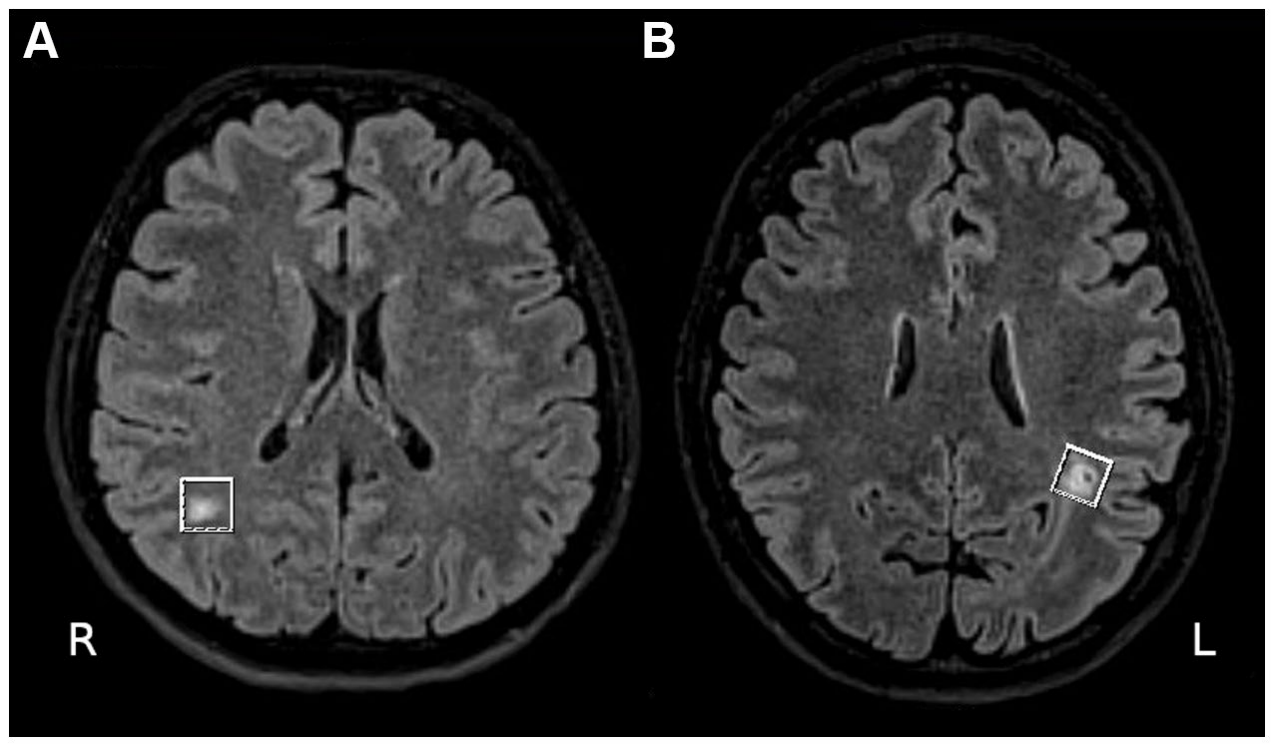
**(A)** A 47-year-old woman with an aura free migraine. The axial fluid-attenuated inversion recovery (FLAIR) MR image shows a large white matter lesion (WML) in the right occipital lobe with normal appearing white matter (NAWM) on the contralateral side. **(B)** A 30-year-old female patient with relapsing–remitting multiple sclerosis. The axial FLAIR MR image shows a large WML in the left supraventricular parietal lobe with NAWM on the contralateral side.

WMLs were defined as hyperintensities on T2-weighted and FLAIR images, without hypointensity on T1-weighted scans, measuring at least 3 mm or larger. Only one WML was investigated in each patient. The investigated WMLs’ largest diameters ranged between 7 and 21 mm and appeared in at least 3 consecutive axial slices on 3D FLAIR images (range between 3 and 10 slices). All investigated WMLs were in the supratentorial deep white matter (Figure 2).

Sagittal T1-weighted images were obtained using a fast low angle shot (FLASH) 2-dimensional (2D) sequence: TR/TE = 300/2.46 milliseconds, flip angle = 88°, 27 slices, slice thickness = 4 mm, 30% interslice gap, FOV = 220 × 220 mm^2^, matrix size = 256 × 320, receiver bandwidth = 330 Hz/pixel.

For T2-weighted images, a turbo spin echo sequence was used: TR/TE = 6000/93 milliseconds, 30 slices, slice thickness = 4 mm, distance factor 20% (0.8 mm gap), FOV = 193 × 220 mm^2^, 280 × 320-pixel matrix, bandwidth = 220 Hz/pixel, number of echo trains = 18. A turbo spin echo sequence was also used for the 3D FLAIR images: TR/TI/TE = 15,710/2750.8/105 milliseconds, 100 slices, slice thickness = 1.5 mm, distance factor 0% (no gap), interleaved slice readout with 2 concatenations, FOV = 220 × 220 mm^2^, 192 × 192-pixel matrix, bandwidth = 400 Hz/pixel, number of echo trains = 14.

Diffusion was determined with a trace-weighted single-shot echo planar 2D imaging sequence: TR/TE = 4000/119 milliseconds, number of slices 20, slice thickness = 3.5 mm, distance factor 0% (no gap), FOV = 188 × 250 mm^2^, 144 × 192 mm^2^ pixel matrix, FOV phase 75%, number of acquisitions 4, *b* values 0, 500, 1,000 s/mm^2^.

Perfusion images were acquired with a single-shot echo planar 2D sequence: TR/TE = 1400/33 milliseconds, flip angle = 68°, 20 slices, slice thickness = 3 mm, distance factor 33%, FOV = 210 × 210 mm^2^, 176 × 176-pixel matrix, bandwidth = 1,150 Hz/pixel, fat saturation switched on. One hundred volumes were consecutively acquired, and contrast agents were administered after acquiring the 20th volume. A Medrad power injector was used for contrast agent and saline administration. A 0.1 mL/body weight amount of gadopentetate dimeglumine (Magnevist, Bayer Schering Pharma AG, Berlin, Germany) was given at a 5 mL/s flow rate, and a 30-ml saline flush was also used for washing at a 5 ml/s flow rate.

Spectroscopy was performed before contrast agent administration to avoid any confounding effects on T1 and T2 relaxations. Before single-voxel ^1^H-MRS acquisition, a voxel of 12 × 12 × 12 mm^3^ was positioned on a preselected WML. Two voxels were placed in every migraine and MS patient: one in the selected WML showing the radiological characteristics of the corresponding disease and one in the contralateral, homotopic, NAWM area without MRI signal abnormality. Two voxels were defined in each healthy subject according to the locations of the voxels of the age-matched migraine patient. Voxels were placed using T2 and 3D FLAIR images to position each voxel. After localized manual shimming and water suppression adjustment, fully relaxed short-echo time proton magnetic resonance spectra (point resolved spectroscopy sequence [PRESS], TR/TE = 6000/30 milliseconds, 128 accumulations, bandwidth = 1,200 Hz, vector size 1,024 points) were acquired. Water suppression was accomplished with a chemical shift-selective sequence pulse. At the end of the ^1^H-MRS acquisition, a reference water signal for the calibration of metabolite concentration was also acquired by turning off the water suppression. After acquiring the metabolite spectra, the water signal T1 and T2 parameters were also determined. T1 was measured using the saturation-recovery method. Six data points were collected, and only the TR was changed between each data point acquisition using the PRESS sequence: TR = 490, 900, 1,400, 2000, 3,000, and 4,000 milliseconds; TE = 30 milliseconds, 1 accumulation, water signal suppression turned off, bandwidth = 2,500 Hz, vector size = 1,024, 4 preparation scans. T2 was obtained by measuring six data points with parameters differing only in echo times using the PRESS sequence: TR = 3,000 milliseconds, TE = 30, 60, 90, 120, 180, and 240 ms, one accumulation, water signal suppression turned off, bandwidth: 2500 Hz, vector size = 1,024, four preparation scans. The total experimental protocol lasted for 45.5 min in the following order: (1) ~28 min for quantitative proton spectroscopy (with manual adjustments); (2) ~2.5 min for T1 measurement; (3) ~3 min for T2 measurement; (4) ~6.5 min for apparent diffusion coefficient (ADC) quantification; (5) ~1 min for T2-weighted images; (6) ~3.5 min for 3D FLAIR; (7) ~2 min for perfusion.

### Data analysis

The T1 relaxation time for each voxel was calculated from the acquired water signals with different repetition times by applying a standard exponential fit:


M=M0∗(1−exp(−TR/T1))

where M is the actual signal intensity, M0 is the signal intensity at thermal equilibrium, and TR is the repetition time.

The T2 relaxation time for each voxel was calculated from the acquired water signals with different echo times, assuming a standard exponential signal decay:


M=M0∗exp(−TE/T2)

where M is the actual signal intensity, M0 is the signal intensity at thermal equilibrium, and TE is the echo time. Curve fittings were carried out on a Siemens Leonardo workstation using Siemens spectroscopy software. Only Fourier transformation and phase correction on the measured signals were applied; no filters or any other corrections were used. The integral of the fitted water signal was used for the T1 and T2 fittings.

To calculate the ADC values, freehand regions of interest (ROIs) were drawn on b0 images on the preselected WMLs. ROIs covered the WMHs selectively. Just like for the spectroscopy, a control area was also measured in the contralateral, homotopic NAWM. Within each ROI, the mean intensities for the b0, b500, and b1000 images were monoexponentially fitted using the following equation:


M=M0∗exp(−b∗ADC)

where M is the measured signal intensity in the presence of diffusion sensitization, M0 is the signal intensity in the absence of diffusion sensitization, b is the b-value, and ADC is the ADC value. Curve fitting and data analysis were performed using Matlab software (The MathWorks, Inc., Natick, MA, USA) for T1, T2, and ADC fittings.

**Figure 3:**
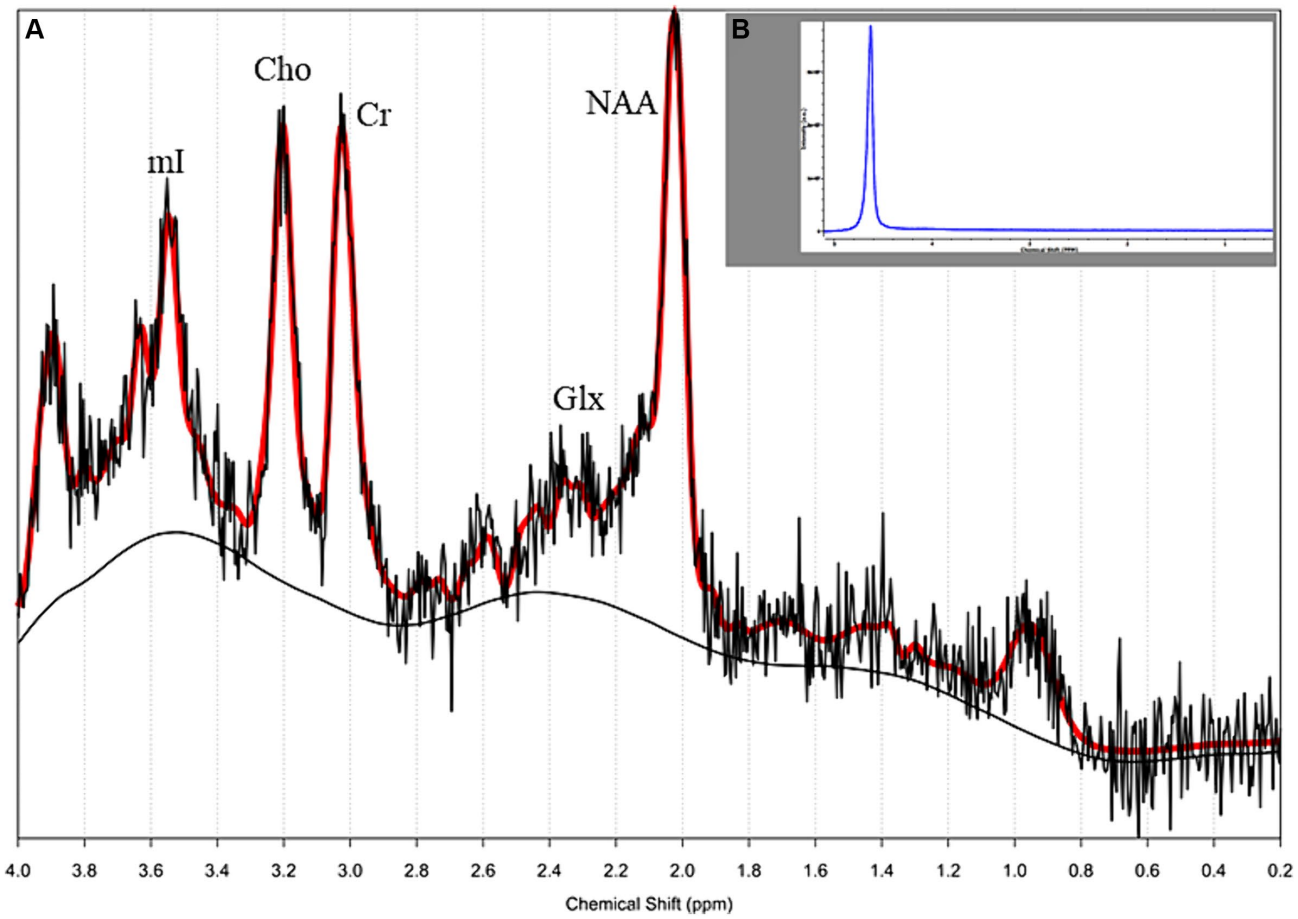
**(A)** Single-voxel proton magnetic resonance spectroscopy (^1^H MRS) of a 30-year-old female patient with relapsing–remitting (RR) multiple sclerosis (MS), (see the lesion on Figure 2B). The morphology of the spectrum looks normal, the concentrations of quantitative metabolites were in the normal range (water suppressed and water-scaled); **(B)** the spectrum of water acquired from the same voxel.

For metabolite quantitative analysis, spectroscopic raw data were postprocessed using the LCModel (Stephen Provencher Inc., Oakville, Ontario, Canada). The concentrations of NAA, glutamate + glutamine + GABA, (Glx), Cho, creatine + phosphocreatine (Cr), and mI were determined (Figure 3).

Perfusion analysis was performed on a Siemens Leonardo Workstation using Siemens Perfusion software (Siemens Medical Solutions, Erlangen, Germany). The relative cerebral blood flow (rCBF) and relative cerebral blood volume (rCBV) were calculated. The arterial input function was determined by an experienced radiologist. Freehand region of interest (ROI) analysis was performed. Lesions with a moderate T2 signal abnormality verified on T2 and FLAIR images could also be identified on the native, slightly T2-and T2*-weighted raw ep2d images measured for perfusion analysis as high-intensity areas. ROIs were drawn on these raw ep2d images; afterwards, these ROIs were copied and used at the same slice of the calculated perfusion maps. The lesions and control areas to be measured as ROIs were selected and verified on T2 and FLAIR images to have a control contralateral white matter area without signal abnormality.

### Statistical analysis

To test normality, the Kolmogorov–Smirnov and Shapiro–Wilk tests were applied. Because the variables did not follow the normal distribution, non-parametric tests were used in subsequent analyses. The acquired MRI variables were compared between the lesional side and the contralateral NAWM of the subjects in each group separately using Mann–Whitney U-tests. The same variables for the lesional and then the contralateral sides were compared among the three subject groups using Kruskal-Wallis tests with *post hoc* analyses. Due to multiple comparisons, *p* values were Bonferroni corrected. Fisher’s exact test was used to examine the difference in the distribution of lobar locations of WMLs between the migraine and MS groups. The ability of intralesional mI to discriminate MS lesions from migraine lesions was assessed by fitting the receiver operating characteristic (ROC) curve and calculating sensitivity, specificity, and area under the ROC curve (AUC). All statistical tests were performed using SPSS Statistics 25.0 software (IBM Corp., Armonk, NY). Results were considered significant (with Bonferroni correction, where applicable) at *p* ≤ 0.05.

## Results

### Comparison between the intralesional and the NAWM voxels

In the migraine group, decreased NAA and creatinine (*p* = 0.016 and *p* < 0.001, respectively), and elevated T2 relaxation time and ADC values (*p* ≤ 0.001) were detected in the WMLs compared to the contralateral NAWM (see data in Table 1).

**Table 1:** Intralesional and contralateral normal-appearing white matter MR voxel findings (mean ± standard deviation).

	Migraine			MS			Control		
	Intralesional	Contralateral NAWM	*p*-value	Intralesional	Contralateral NAWM	*p*-value	Intralesional	Contralateral NAWM	*p*-value
NAA (mmol/L)	7.8 ± 1.1	8.9 ± 1.0	0.016	6.6 ± 1.1	8.1 ± 0.8	<0.001	8.7 ± 0.9	8.7 ± 1.3	ns
Glx (mmol/L)	8.0 ± 1.4	8.6 ± 2.0	ns	6.8 ± 1.5	8.0 ± 1.9	ns	8.3 ± 2.1	8.2 ± 2.1	ns
Cho (mmol/L)	1.5 ± 0.3	1.7 ± 0.3	ns	1.6 ± 0.4	1.5 ± 0.2	ns	1.6 ± 0.3	1.7 ± 0.2	ns
Cr (mmol/L)	4.8 ± 0.5	5.6 ± 0.4	<0.001	5.1 ± 0.6	5.5 ± 0.1	ns	5.6 ± 0.4	6.0 ± 1.0	ns
mI (mmol/L)	4.6 ± 0.9	4.5 ± 0.9	ns	6.4 ± 1.3	5.9 ± 0.9	ns	5.1 ± 1.3	4.7 ± 1.1	ns
T1 (ms)	1090.4 ± 137.8	1057.6 ± 111.5	ns	1203.7 ± 142.6	1030.3 ± 78.2	<0.001	1007.3 ± 86.2	1029.4 ± 99.7	ns
T2 (ms)	83.2 ± 11.6	70.9 ± 6.3	0.001	88.5 ± 16.2	70.7 ± 4.8	<0.001	69.7 ± 8.7	71.2 ± 7.2	ns
rCBF	47.3 ± 22.2	67.5 ± 31.8	ns	49.7 ± 38.3	70.2 ± 41.6	ns	60.3 ± 30.3	58.9 ± 33.3	ns
rCBV	197.2 ± 73.5	256.2 ± 96.6	ns	210.5 ± 114.5	250.0 ± 100.3	ns	206.0 ± 52.7	207.4 ± 66.4	ns
ADC (×10^−4^ mm^2^/s)	9.7 ± 1.7	6.6 ± 0.8	<0.001	11.2 ± 2.3	6.8 ± 0.4	<0.001	6.2 ± 0.6	6.6 ± 0.6	ns

MR, magnetic resonance; NAWM, normal appearing white matter; MS, multiple sclerosis; NAA, N-acetyl aspartate; Glx, glutamate and glutamine; Cho, choline compounds; Cr, creatine/phosphocreatine; mI, myo-inositol; T1, T1 relaxation time; T2, T2 relaxation time; CBF, cerebral blood flow; CBW, cerebral blood volume; ADC, apparent diffusion coefficient.

Similarly, in the MS group, decreased NAA and elevated T2 relaxation time and ADC values (*p* < 0.001 in all cases) were found in the WMLs compared to the contralateral side. In addition, elevated T1 values were also observed in MS WMLs (*p* < 0.001), while Cr values did not differ significantly between the two sides (see details in Table 1).

No difference was observed between the two hemispheres of the control subjects in any MRI variables. Perfusion variables (rCBF and rCBV) did not show any difference between the intralesional and the contralateral sides in any groups.

### Comparison among the three subject groups

Fisher’s exact test indicated that the distribution of lobar location of the large WMLs were not different between our two patient groups, regardless of whether the lobes were considered bilateral structures or whether the left and right hemispheric lobes were considered separately (*p* = 0.697 and *p* = 0.954, respectively; see distribution in Table 2). On the lesional side, significant differences were shown in NAA, Cr, mI, T1, T2 relaxation times, and ADC values among the 3 patient groups (*p* ≤ 0.001 in all cases). T1, T2 relaxation times, and ADC values followed a similar trend, with the lowest values observed in controls and the highest in MS patients (Table 3). In *post hoc* tests, migraine patients showed significantly higher NAA and lower mI and T1 values than MS patients (*p* = 0.033, *p* = 0.001, and *p* = 0.029, respectively, see details in Table 3). Moreover, migraineurs had decreased creatine and increased T2 relaxation time and ADC values compared to the control subjects (*p* ≤ 0.001 in all cases, Table 3). The MS group showed elevated mI (*p* = 0.035), T1, T2 relaxation time, and ADC (*p* ≤ 0.001), and reduced NAA (*p* < 0.001) values compared to the control group (Table 3). The ROC analysis for intralesional mI indicated that MS lesions can be differentiated from migraine lesions with a sensitivity of 86.7% and a specificity of 82.4% when using an optimal cutoff point of 5.224 (i.e., the cutoff point nearest to the upper left corner of the ROC space); AUC = 0.878.

**Table 2:** Lobar distribution of the white matter large lesions in patients with migraine and multiple sclerosis (MS).

	Right side				Left side				Total
	Frontal	Parietal	Temporal	Occipital	Frontal	Parietal	Temporal	Occipital	
Migraine	6	1	1	1	5	2	–	1	17
MS	5	3	–	–	4	2	–	1	15

**Table 3:** Group comparisons.

		**Migraine vs. MS**	**Migraine vs. control**	**MS vs. control**
Intralesional	NAA (mmol/L)	7.8 ± 1.1 vs. 6.6 ± 1.1	7.8 ± 1.1 vs. 8.7 ± 0.9	6.6 ± 1.1 vs. 8.7 ± 0.9
		*p* = 0.033	ns	*p* < 0.001
	Cr (mmol/L)	4.8 ± 0.5 vs. 5.1 ± 0.6	4.8 ± 0.5 vs. 5.6 ± 0.4	5.1 ± 0.6 vs. 5.6 ± 0.4
		ns	*p* = 0.001	*p* = 0.055
	mI (mmol/L)	4.6 ± 0.9 vs. 6.4 ± 1.3	4.6 ± 0.9 vs. 5.1 ± 1.3	6.4 ± 1.3 vs. 5.1 ± 1.3
		*p* = 0.001	ns	*p* = 0.035
	T1 (ms)	1090.4 ± 137.8 vs. 1203.7 ± 1 42.6	1090.4 ± 137.8 vs. 1007.3 ± 86.2	1203.7 ± 142.6 vs. 1007.3 ± 86.2
		*p* = 0.029	ns	*p* < 0.001
	T2 (ms)	83.2 ± 11.6 vs. 88.5 ± 16.2	83.2 ± 11.6 vs. 69.8 ± 8.7	88.5 ± 16.2 vs. 69.8 ± 8.7
		ns	*p* = 0.003	*p* = 0.001
	ADC (x10^−4^ mm^2^/s)	9.7 ± 1.7 vs. 11.2 ± 2.3	9.7 ± 1.7 vs. 6.3 ± 0.6	11.2 ± 2.3 vs. 6.3 ± 0.6
		ns	*p* < 0.001	*p* < 0.001
Contralateral NAWM	Cho (mmol/L)	1.7 ± 0.3 vs. 1.5 ± 0.2	1.7 ± 0.3 vs. 1.7 ± 0.2	1.5 ± 0.2 vs. 1.7 ± 0.2
		*p* = 0.049	ns	ns
	mI (mmol/L)	4.5 ± 0.9 vs. 5.9 ± 0.9	4.5 ± 0.9 vs. 4.7 ± 1.1	5.9 ± 0.9 vs. 4.7 ± 1.1
		*p* = 0.001	ns	*p* = 0.018

Only significant differences in Kruskal-Wallis tests are presented in the table (mean ± standard deviation). *p*-values are shown after the Bonferroni correction. NAWM, normal appearing white matter; MS, multiple sclerosis; NAA, *N*-acetyl aspartate; Cr, creatine + phosphocreatine; mI, myo-inositol; T1, T1 relaxation time; T2, T2 relaxation time; ADC, apparent diffusion coefficient; Cho, choline compounds.

In the contralateral NAWM, lower mI values were observed in migraine patients compared to MS patients, while choline values showed only a trend (*p* = 0.001 and *p* = 0.05, respectively, Table 3). MS patients showed higher mI values than control subjects in the NAWM (*p* = 0.018). No significant difference was found in any values of the normal-appearing side contralateral to the lesion between the migraine and control groups (Table 3). None of the PWI variables were different in any comparisons among the three groups.

## Discussion

In this study, we investigated and compared the WMLs of migraine and MS patients using advanced MRI techniques in patients with normal-appearing T1-weighted MR imaging. In both disorder groups, elevated T2 relaxation time, ADC values, and decreased NAA values were found in the intralesional white matter compared to the contralateral NAWM, while there was no difference between the hemispheres in the control subjects. Migraine patients had the lowest intralesional Cr and mI values among the three groups, while patients with MS showed the highest intralesional T1, T2 relaxation times, and ADC and mI values. In the contralateral NAWM, the same trend of mI changes was observed in migraineurs and MS patients.

Although the pathophysiology of the WMLs in the two disorders is different, differentiation of these comorbid diseases with conventional MRI is challenging in several cases (Liu et al., 2013). In general, migraine-related WMLs are smaller and fewer than those seen in MS and can progress with disease duration, based on our recent longitudinal study (Erdélyi-Bótor et al., 2015). The origin of WMLs in migraine is supposed to be a microvascular ischemic pathomechanism due to attack-related oligemia and focal hypoperfusion (Kruit et al., 2006; Dodick and Roarke, 2008; Ersoy et al., 2020), while MS lesions are the result of the blood–brain barrier disruption due to a complex mixture of primary degenerative processes, involvement of innate immunity, CD4+, CD8+ T cells, and B cells (Rahmanzadeh et al., 2018).

Advanced MRI techniques may help us better differentiate the underlying mechanisms. The prolonged T1 and T2 relaxation times and elevated ADC values suggest an increased extracellular water fraction and increased diffusivity in both migraine and MS lesions, probably due to tissue damage (Pagani et al., 2008; Zhu et al., 2011). The values were higher in the MS group, indicating that the tissue damage is more severe than in migraine. ^1^H-MRS can provide more information about the concentration of major neurotransmitters and metabolites (Moore, 1998; Heckova et al., 2022; Nikolova and Schwedt, 2022; Lipka et al., 2023). Recent studies using 7 T MRS imaging reported that metabolic abnormalities in the NAWM and cortical gray matter were associated with disability (Heckova et al., 2022), and the measure of mI may serve as an early biomarker of lesion development in MS patients (Lipka et al., 2023).

In line with the above-mentioned results, low NAA levels observed in both groups (MS < migraine) may refer to decreased axonal viability and function and cell loss in the white matter, while decreased Cr values (migraine < MS) can reflect tissue degeneration with low cellularity and impaired mitochondrial functioning (Moore, 1998; Barkhof and van Walderveen, 1999; Mader et al., 2008). The lower intralesional Glx concentration (MS < migraine) is not significant but may represent tissue damage. It contains the excitatory neurotransmitter glutamate and glutamine, and the inhibitory neurotransmitter GABA in a small proportion (Moore, 1998; Minati et al., 2010; Ellingson et al., 2019). Normal level of Cho and the lack of lactate peak do not indicate active myelin breakdown, remyelination, or presence of anaerobic glycolysis in any of the studied groups. The missing high level of Cho (astrocytosis in active demyelination) and mI (astrogliosis, T1-weighted image hypointensity, black hole) in MS is likely the consequence of the remission phase of patient and the low EDSS score (Granziera et al., 2021). Higher mI levels in MS patients compared to migraineurs and the control group may indicate a reactive astrocytic gliosis in chronic MS lesions (Moore, 1998; Barkhof and van Walderveen, 1999; Mader et al., 2008; Granziera et al., 2021).

Beyond the statistically significant findings, the tables show similar intralesional tendencies toward pathology in both migraineurs and MS patients. Taking into consideration the progressive nature of lesion formation (Erdélyi-Bótor et al., 2015; Multiple and Pathology, 2018), the locations of the WMLs are quite similar in the groups; only the optic nerve demyelination, the large hemispheric tumefactive demyelination, the black hole, the lesion with ring-like contrast agent enhancement, and the spinal cord lesion can be detected in MS (Klistorner et al., 2017). Behind the similarities, the lesion pathology contains oxidative stress (Erdélyi-Bótor et al., 2017; Multiple and Pathology, 2018; Gross et al., 2021) and autoimmunity (Arumugam and Parthasarathy, 2016; Arumugam and Narayan, 2019; Tobin et al., 2021; Bagherzadeh-Fard et al., 2023). Oxidation is a normal and necessary process that takes place in the human body. Oxidative stress occurs when there is an imbalance between free radical activity and antioxidant activity. When there are more free radicals present than can be kept in balance by antioxidants, the free radicals can start doing damage to fatty tissue, DNA, and proteins in the body. Proteins, lipids, and DNA make up a large part of the body and brain, as well, so that damage can lead to a vast number of progressive diseases over time (Hajam et al., 2022). Migraine and systemic autoimmune diseases (Sjögren’s syndrome, systemic lupus erythematosus, antiphospholipid syndrome, and other diffuse connective tissue diseases) are 2-3-fold more common in women, and various studies have reported an association between the two pathologies. Endothelial dysfunction is the only alteration that is common among all these disorders (Cavestro and Ferrero, 2018). Due to the comorbidity between MS and migraine, radiological separation is difficult when an MS patient suffers from migraine attacks (Kister et al., 2010).

The present study has some limitations, such as the small patient population due to the strict inclusion criteria and the fact that the voxels contained not just the WMLs but also perilesional white matter in different proportions. The small sample size does not allow us to control for potential confounding factors or investigate the possible differences between the migraine subgroups. The ROC curve was fitted on the same data used for the assessment of sensitivity and specificity values; therefore, the reported values may be optimistically biased.

In conclusion, our multimodal study showed that tissue damage is detectable in both diseases. Although the injury seemed to be more severe in MS than migraine, we could not clearly differentiate the two diseases using advanced MRI techniques; however, the small sample size prevents us from drawing general conclusions.

## Data availability statement

The raw data supporting the conclusions of this article will be made available by the authors, without undue reservation.

## Ethics statement

The studies involving humans were approved by Regional Research Ethics Committee of the Clinical Center, Pécs. The studies were conducted in accordance with the local legislation and institutional requirements. The participants provided their written informed consent to participate in this study.

## Author contributions

FJ: Writing – original draft, Writing – review & editing, Conceptualization, Data curation, Formal analysis, Investigation, Methodology, Project administration. GK-J: Data curation, Investigation, Project administration, Validation, Writing – original draft, Writing – review & editing. HK: Writing – original draft, Writing – review & editing. GP: Conceptualization, Formal analysis, Funding acquisition, Methodology, Resources, Software, Supervision, Validation, Writing – original draft, Writing – review & editing. GO: Conceptualization, Formal analysis, Funding acquisition, Methodology, Resources, Software, Supervision, Validation, Writing – original draft, Writing – review & editing. EB: Data curation, Writing – original draft, Writing – review & editing. RR: Data curation, Writing – original draft, Writing – review & editing. AT: Data curation, Writing – original draft, Writing – review & editing. KE: Data curation, Writing – original draft, Writing – review & editing. DK: Conceptualization, Data curation, Visualization, Writing – original draft, Writing – review & editing. ZP: Conceptualization, Data curation, Funding acquisition, Investigation, Methodology, Project administration, Resources, Supervision, Visualization, Writing – original draft, Writing – review & editing.
